# Supplementary material for: How self-governance willingness and participation efficacy shape residents’ satisfaction with urban public services: Evidence from neighborhood renewal in Hangzhou, China
Source: PLoS One. 2026 Jan 23;21(1):e0341177. doi: 10.1371/journal.pone.0341177 (PMC12829847; doi:10.1371/journal.pone.0341177)
Supplement: S1 Table — (PDF) [file pone.0341177.s002.pdf]

Table S1 presents the results of the Harman's single-factor test for common-method variance (CMV). The unrotated exploratory factor analysis extracted six factors with eigenvalues greater than 0.5. The first factor has an eigenvalue of 1.821 and explains 30.4% of the total variance, which is well below the conventional 40% threshold commonly used to indicate serious CMV. The remaining factors jointly account for the remaining 69.6% of the variance. These results confirm that no single factor dominates the covariance structure, suggesting that CMV is unlikely to bias the study's findings.

Table S1. Harman's single-factor test

Factor	Eigenvalue	Difference	Proportion	Cumulative
Factor1	1.821	0.235	0.304	0.304
Factor2	1.587	0.811	0.264	0.568
Factor3	0.776	0.109	0.129	0.697
Factor4	0.666	0.030	0.111	0.808
Factor5	0.636	0.121	0.106	0.914
Factor6	0.515	.	0.086	1.000
