# Supplementary material for: How self-governance willingness and participation efficacy shape residents’ satisfaction with urban public services: Evidence from neighborhood renewal in Hangzhou, China
Source: PLoS One. 2026 Jan 23;21(1):e0341177. doi: 10.1371/journal.pone.0341177 (PMC12829847; doi:10.1371/journal.pone.0341177)
Supplement: S2 Table — (PDF) [file pone.0341177.s003.pdf]

Table S2 presents robustness checks using alternative city-level instruments. Distance2 measures the distance from each neighborhood's centroid to the Hangzhou Municipal Government office, and Distance3 measures the distance to the city's largest railway transportation hub. In the first-stage regressions, both instruments are positively associated with residents' willingness to engage in self-governance, with coefficients ranging from 0.005 to 0.006, all significant at the 1% level. The Kleibergen–Paap rk LM (51.449–54.215) and Kleibergen–Paap rk Wald F (68.279–79.096) statistics exceed conventional thresholds, confirming the instruments' validity and ruling out weak identification concerns.

Columns (1) and (3) report baseline estimates without control variables, while columns (2) and (4) include the full set of control variables. In the second stage, the estimated coefficients of self-governance willingness remain positive (ranging from 0.114 to 0.221) and statistically significant across all model specifications. These consistent results demonstrate that replacing the original provincial-level instrument with city-level measures and varying control specifications does not alter the magnitude or significance of the estimated effects, thereby reinforcing the robustness and validity of the identification strategy.

**Table S2. Robustness checks using alternative city-level instruments**

| | **Public Services Satisfaction** | | | |
|---|---|---|---|---|
| | **(1)** | **(2)** | **(3)** | **(4)** |
| Panel A: Second stage | | | | |
| Self-governance willingness | 0.201** | 0.114* | 0.221*** | 0.130** |
| | (2.53) | (1.71) | (2.74) | (1.98) |
| Panel B: First stage | | | | |
| Distance2 | 0.005*** | 0.005*** | | |
| | (8.26) | (8.75) | | |
| Distance3 | | | 0.005*** | 0.006*** |
| | | | (8.31) | (8.89) |
| *Kleibergen-Paap rk LM* | 51.449 | 52.355 | 51.749 | 54.215 |
| *Kleibergen-Paap rk Wald F* | 68.279 | 76.547 | 68.992 | 79.096 |
| Control variables | No | Yes | No | Yes |
| District FE | Yes | Yes | Yes | Yes |
| Street-level FE | Yes | Yes | Yes | Yes |
| N | 2202 | 2202 | 2202 | 2202 |
| $R^2$ | 0.277 | 0.460 | 0.244 | 0.443 |

Notes: *, **, and *** indicate statistical significance at the 10%, 5%, and 1% levels, respectively. All models include district and street fixed effects. Robust t-statistics (in parentheses) are computed using heteroskedasticity-consistent standard errors. Following standard practice in instrumental variable (IV) regressions, the uncentered $R^2$ is reported.
