# Supplementary material for: How self-governance willingness and participation efficacy shape residents’ satisfaction with urban public services: Evidence from neighborhood renewal in Hangzhou, China
Source: PLoS One. 2026 Jan 23;21(1):e0341177. doi: 10.1371/journal.pone.0341177 (PMC12829847; doi:10.1371/journal.pone.0341177)
Supplement: Attachment — Submitted filename: Response to Reviewers.docx [file pone.0341177.s005.docx]

**Response to Editor**

1. Please ensure that your manuscript meets PLOS ONE's style requirements, including those for file naming. The PLOS ONE style templates can be found at

https://journals.plos.org/plosone/s/file?id=wjVg/PLOSOne_formatting_sample_main_body.pdf and

<https://journals.plos.org/plosone/s/file?id=ba62/PLOSOne_formatting_sample_title_authors_affiliations.pdf>.

Thank you for the reminder. We have updated the manuscript and all associated files to conform to PLOS ONE style and the provided templates. Specifically, we have adopted the required file-naming conventions; formatted the title/authors/affiliations and main text per the templates; verified figure and table numbering, captions, and in-text callouts; standardized reference formatting. Please let us know if any further adjustments are needed.

2. Please provide additional details regarding participant consent. In the ethics statement in the Methods and online submission information, please ensure that you have specified (1) whether consent was informed and (2) what type you obtained (for instance, written or verbal, and if verbal, how it was documented and witnessed). If your study included minors, state whether you obtained consent from parents or guardians. If the need for consent was waived by the ethics committee, please include this information.

If you are reporting a retrospective study of medical records or archived samples, please ensure that you have discussed whether all data were fully anonymized before you accessed them and/or whether the IRB or ethics committee waived the requirement for informed consent. If patients provided informed written consent to have data from their medical records used in research, please include this information.

Thank you for raising this. We have further strengthened the Ethics/Consent statement in both the Methods and the online submission to fully address PLOS ONE’s requirements: (i) consent was informed and obtained verbally prior to each interview; enumerators read a standardized script and recorded consent on the questionnaire cover sheet before proceeding; (ii) all respondents were adult homeowners (≥18 years)—no minors were enrolled; (iii) participation was voluntary, with the right to decline or discontinue at any time without consequence; (iv) no medical records or biospecimens were used; all survey data were de-identified at the point of collection and contain no personally identifying information; (v) data were stored securely with access limited to the research team and are reported only in aggregate; and (vi) no consent waiver was sought or granted. Fieldwork consisted of one-to-one, face-to-face interviews using paper questionnaires conducted on September 15, 20–22, 2024.

3. Thank you for stating in your Funding Statement:

“This research is supported by the 2020 General Project of the National Social Science Fund of China (20BZZ040).”

Please provide an amended statement that declares *all* the funding or sources of support (whether external or internal to your organization) received during this study, as detailed online in our guide for authors at http://journals.plos.org/plosone/s/submit-now.  Please also include the statement “There was no additional external funding received for this study.” in your updated Funding Statement.

Please include your amended Funding Statement within your cover letter. We will change the online submission form on your behalf.

Thank you for the guidance. We have amended the Funding Statement as requested and included it verbatim in the cover letter:

Funding: This work was supported by the National Social Science Fund of China (Grant No. 20BZZ040). There was no additional external funding received for this study.

4. Thank you for stating the following financial disclosure:

“This research is supported by the 2020 General Project of the National Social Science Fund of China (20BZZ040).”

Please state what role the funders took in the study.  If the funders had no role, please state: "The funders had no role in study design, data collection and analysis, decision to publish, or preparation of the manuscript."

If this statement is not correct you must amend it as needed.

Please include this amended Role of Funder statement in your cover letter; we will change the online submission form on your behalf.

Thank you for the request. We have added the following statement to the cover letter and kindly invite you to update the online submission form on our behalf:

Role of the funders: The National Social Science Fund of China (Grant No. 20BZZ040) provided financial support only and had no role in study design, data collection and analysis, decision to publish, or preparation of the manuscript. The authors had full independence and full access to all data.

5. We note that your Data Availability Statement is currently as follows: [All relevant data are within the manuscript and its Supporting Information files.]

Please confirm at this time whether or not your submission contains all raw data required to replicate the results of your study. Authors must share the “minimal data set” for their submission. PLOS defines the minimal data set to consist of the data required to replicate all study findings reported in the article, as well as related metadata and methods (https://journals.plos.org/plosone/s/data-availability#loc-minimal-data-set-definition).

For example, authors should submit the following data:

- The values behind the means, standard deviations and other measures reported;

- The values used to build graphs;

- The points extracted from images for analysis.

Authors do not need to submit their entire data set if only a portion of the data was used in the reported study.

If your submission does not contain these data, please either upload them as Supporting Information files or deposit them to a stable, public repository and provide us with the relevant URLs, DOIs, or accession numbers. For a list of recommended repositories, please see https://journals.plos.org/plosone/s/recommended-repositories.

If there are ethical or legal restrictions on sharing a de-identified data set, please explain them in detail (e.g., data contain potentially sensitive information, data are owned by a third-party organization, etc.) and who has imposed them (e.g., an ethics committee). Please also provide contact information for a data access committee, ethics committee, or other institutional body to which data requests may be sent. If data are owned by a third party, please indicate how others may request data access.

We confirm that the submission includes the complete minimal dataset. We have uploaded a single de-identified respondent-level file as Supporting Information (S1 Dataset) containing all variables and observations used in the analyses, with header names matching the variable definitions in the manuscript. This file provides the values underlying all coefficients, standard errors, figures, and summary statistics. No additional files or access permissions are required.

6. Thank you for stating the following in the Acknowledgments Section of your manuscript:

“This research is supported by the 2020 General Project of the National Social Science Fund of China (20BZZ040).”

We note that you have provided additional information within the Acknowledgements Section that is not currently declared in your Funding Statement. Please note that funding information should not appear in the Acknowledgments section or other areas of your manuscript. We will only publish funding information present in the Funding Statement section of the online submission form.

Please remove any funding-related text from the manuscript and let us know how you would like to update your Funding Statement. Currently, your Funding Statement reads as follows:

“This research is supported by the 2020 General Project of the National Social Science Fund of China (20BZZ040).”

Please include your amended statements within your cover letter; we will change the online submission form on your behalf.

Thank you for the guidance. We have removed all funding-related text from the Acknowledgments section. Please update our Funding Statement as follows:

Funding: This work was supported by the National Social Science Fund of China (Grant No. 20BZZ040). There was no additional external funding received for this study.

Role of the funders: The National Social Science Fund of China (Grant No. 20BZZ040) provided financial support only and had no role in study design, data collection and analysis, decision to publish, or preparation of the manuscript. The authors had full independence and full access to all data.

7. If the reviewer comments include a recommendation to cite specific previously published works, please review and evaluate these publications to determine whether they are relevant and should be cited. There is no requirement to cite these works unless the editor has indicated otherwise.

Thank you for the reminder. We reviewed all reviewer reports and did not find any requests to cite specific previously published works. Should such recommendations arise, we will evaluate their relevance and add citations where appropriate.

Additional Editor Comments (if provided):

1. The manuscript’s theoretical contribution is weak, offering limited novelty beyond existing participatory governance and satisfaction frameworks.

We appreciate the reviewer’s concern. The revision clarifies a distinct contribution by advancing a resident-centered account of participatory governance that theoretically differentiates self-governance willingness (orientation to autonomous governance) from participation efficacy (perceived effectiveness within institutional channels) and specifies asymmetric effects, efficacy mitigates perceived neighborhood disparities and raises satisfaction, whereas willingness can heighten disparity sensitivity absent institutional responsiveness, suppressing its direct positive association with satisfaction. We formalize a parsimonious causal pathway—resident agency → perceived neighborhood disparities → satisfaction—thereby making comparative fairness an explicit mediating mechanism rather than an implicit assumption in prior work, and we identify institutional responsiveness (procedurally binding participation in legally structured programs) as a boundary condition under which willingness translates into higher satisfaction.

To render these advances transparent, we streamlined the Theoretical Framework, added a concise conceptual model mapping these relations, and aligned hypotheses accordingly. The empirical sections then corroborate the propositions via IV identification, mediation centered on perceived disparities, and heterogeneity analyses, demonstrating that the manuscript extends prevailing frameworks by specifying distinct components of agency, a testable mediating channel, and clear institutional contingencies.

2. The research gap is not sharply defined, reducing the strength of the originality claim.

Thank you for this constructive point. We have tightened the articulation of the research gap and made our contribution explicit. The Introduction now closes with a three-part gap statement: (i) the paucity of micro-level causal evidence on distinct dimensions of resident agency—self-governance willingness vs. participation efficacy—and their effects on satisfaction; (ii) limited knowledge of how institutionalized participatory arrangements in China’s legally structured urban renewal shape perceived neighborhood quality and procedural/distributive fairness, including the unexamined mediating role of perceived neighborhood disparities; and (iii) a literature dominated by small-N or normative accounts lacking credible identification. We then map each of our four research questions and hypotheses directly to these gaps and address them using a large resident-level dataset (n = 2,202 across 36 neighborhoods), an IV design for identification, mediation tests centered on perceived disparities, and heterogeneity analyses. These revisions clarify what is missing, why it matters, and how our study fills it, thereby strengthening the originality claim.

3. The case selection of Hangzhou lacks a compelling analytical justification and discussion of generalizability.

We appreciate this comment. The revision now provides a clearer, theory-driven rationale for selecting Hangzhou (Introduction, Section 1). Hangzhou’s old-neighborhood renewal is among China’s largest and most institutionalized participatory programs (≈1,000 projects completed by 2023), and it pioneers procedurally binding participation via the Property Law’s “double two-thirds” rule (≥2/3 to initiate and ≥2/3 to approve plans), thereby embedding substantive public deliberation in renewal decisions. This combination of scale and formal participatory requirements makes Hangzhou an analytically rich setting to observe variation in self-governance willingness and participation efficacy at neighborhood level. We therefore frame Hangzhou as a “most-likely” case for detecting effects of resident engagement on satisfaction—if participatory governance matters, it should be observable here.

We also add an explicit discussion of generalizability (Discussion, Section 6). While Hangzhou is ahead in institutionalizing participation, many Chinese cities are adopting similar renewal programs with participatory elements, making the mechanisms we study relevant to comparable urban contexts. We distinguish what is likely to generalize (e.g., the importance of aligning governance mechanisms with residents’ autonomy preferences and the pathway from participation efficacy to satisfaction via perceived disparities) from what is context-specific (e.g., the magnitude of effects in smaller cities or locales with weaker institutional support). We further note (Limitations, Section 6.2) that Hangzhou’s relative affluence and administrative capacity may not represent all settings. In sum, the manuscript now justifies the Hangzhou case on analytical grounds and clarifies the scope conditions under which the findings are most likely to travel.

4. The operationalization of key constructs with binary indicators oversimplifies complex concepts and may compromise validity.

We appreciate this concern. As detailed in the revision, self-governance willingness and participation efficacy are modeled as latent constructs estimated via exploratory factor analysis (EFA) from multiple, theory-driven binary indicators rather than single dichotomies. For self-governance willingness, three responsibility-allocation items (decision-making, performance evaluation, property-management selection) load on a single factor (eigenvalue = 1.585; variance explained = 52.8%; KMO = 0.612; Bartlett’s χ² = 527.210, p < 0.01). For participation efficacy, three items (pre-decision consultation, process supervision, feedback responsiveness) load on a single factor (eigenvalue = 1.814; variance explained = 60.5%; KMO = 0.648; Bartlett’s χ² = 1027.199, p < 0.01). All models use continuous factor scores, preserving gradations in underlying attitudes despite dichotomous item responses.

To assess sensitivity to operationalization, we (i) reconstructed both constructs as additive indices and (ii) re-estimated models using the original five-point Likert outcome for public-service satisfaction. Across these alternatives—and with the full covariate set plus district and street fixed effects—the substantive conclusions are unchanged (Table 7): self-governance willingness remains positive and significant (coefficients 0.754–0.980, p < 0.01), participation efficacy remains positive and significant (coefficients 0.362–0.501, p < 0.01), and perceived neighborhood disparity is consistently negative. Taken together—transparent construct design, a validated factor structure, continuous factor scoring, and convergent robustness evidence—these steps directly address measurement-validity concerns and indicate that our results are not artifacts of dichotomization or specific coding choices.

5. The instrumental variable choice—especially distance to the provincial government—has weak conceptual justification and questionable exclusion restriction validity.

Thank you for raising this point. As detailed in our response to Reviewer 2, Comment 2, we have substantially strengthened both the conceptual rationale and empirical validation of our IV strategy.

6. Dichotomizing the dependent variable causes loss of information and statistical precision.

As detailed in our response to Reviewer 2, Comment 3, we directly assess sensitivity to dichotomization. Beyond the binary specification (reported for interpretability), we re-estimate all models using the original five-point Likert outcome and, in parallel, reconstruct the key constructs as additive indices. With the full covariate set and district/street fixed effects, results are substantively unchanged (Table 7): self-governance willingness and participation efficacy remain positive and statistically significant, and perceived neighborhood disparity remains negative. These convergent checks indicate that our inferences are not artifacts of dichotomization and that any information loss does not alter the substantive conclusions.

7. Mediation analysis may suffer from endogeneity between the mediator and outcome, undermining causal claims.

We agree that mediation results warrant caution given potential endogeneity between the mediator (perceived neighborhood disparity) and the outcome (satisfaction). Importantly, our causal claims pertain to resident agency—self-governance willingness and participation efficacy—identified via 2SLS/IV; the mediation analysis is used to deepen interpretation, not to establish additional causality.

Methodologically, we include a rich covariate set and district/street fixed effects and follow the Baron–Kenny three-equation approach with Sobel tests. Nonetheless, as the reviewer notes, these steps cannot guarantee mediator exogeneity. We therefore explicitly acknowledge possibilities of reverse causality (e.g., higher satisfaction reducing perceived disparities) and omitted common causes (e.g., unmeasured governance quality). Consistent with this, the revision (Discussion Section 6.2) clarifies that relationships involving the mediator are interpreted as associational. We have softened language from “serves as a mediating mechanism” to “appears to mediate” or “is consistent with a mediating role.”

We also note that the observed patterns (Tables 5–6)—participation efficacy associated with lower perceived disparities and higher satisfaction; willingness showing a suppression pathway via disparities—are theoretically coherent and robust across specifications, but we refrain from causal assertions for the mediator. We suggest future panel or experimental designs (e.g., pre/post neighborhood interventions or information treatments) to test the mediation pathway more conclusively.

In sum, our identification strategy for resident agency is causal (IV), while the mediation analysis is presented as interpretive and consistent with theory, with explicit caveats to avoid over-claiming.

8. The discussion section offers limited theoretical engagement and critical interpretation beyond restating results.

Thank you for this helpful comment. We have substantially revised Section 6 (Discussion) to move beyond a summary of results and provide a theoretically grounded, critical interpretation. Conceptually, we sharpen the resident-centered account by distinguishing self-governance willingness (orientation to autonomous governance) from participation efficacy (perceived effectiveness within institutional channels) and by specifying asymmetric effects consistent with participatory-governance and procedural-justice frameworks. We link the identified pathway—resident agency → perceived neighborhood disparities → satisfaction—to relative deprivation theory, showing why efficacy tends to reduce perceived disparities and elevate satisfaction, whereas willingness can heighten disparity sensitivity absent institutional responsiveness. We also read the IV estimates (Table 4) through empowerment theory rather than technocratic delivery, and we explicitly treat the mediation evidence (Tables 5–6) as interpretive rather than causal.

To deepen critical engagement, the Discussion now contrasts symbolic versus substantive participation, explains how Hangzhou’s “double two-thirds” consent rule and multi-actor architecture mitigate tokenism, and delineates scope conditions under which resident agency is most likely to translate into higher satisfaction. We interpret heterogeneous effects across income, hukou, housing area, and ownership (Tables 8–11) as distributional contingencies rather than uniform gains, and we temper claims where subgroup estimates are imprecise. Finally, we foreground robustness (Table 7) as supporting evidence that the theoretical mechanisms—not measurement choices—drive the findings. Taken together, these revisions provide a clearer dialogue with theory, articulate boundary conditions and alternative explanations, and offer a critical reading of what the results mean beyond the coefficients, thereby directly addressing the editor’s concern.

**Response to Reviewer #1**

Reviewer #1: 1. The manuscript presents a timely and well-executed study on how self-governance willingness and participation efficacy influence satisfaction with urban public services in Hangzhou. The introduction is well-structured and clearly identifies the research gap by shifting focus from government-centric to resident-centered governance. The literature review is rich and cites relevant theoretical and empirical studies; however, further inclusion of global comparative studies on participatory governance would enhance the contextual breadth.

We sincerely thank the reviewer for this valuable suggestion. In the revised manuscript, we have expanded the literature review to include a wider range of influential global comparative studies on participatory governance, thereby broadening the international scope and contextual depth of our analysis. The revised section situates grassroots participatory governance within the global shift toward participatory decision-making and deliberative democracy, integrating both theoretical perspectives—such as citizenship and institutional participation—and cross-national empirical evidence. We draw on studies from advanced democracies (e.g., the United States and the United Kingdom), where active citizen participation has shaped state reform and improved democratic quality, as well as from developing countries, where evidence, though positive, remains limited in scope and methodological rigor. This discussion also positions the Chinese experience within the global context, outlining its transition from symbolic participation to the institutionalization of participatory governance through consultative democracy platforms, participatory budgeting, and localized mechanisms. We believe these revisions address the reviewer’s concern and strengthen the theoretical positioning and comparative relevance of our study within the international scholarship on participatory governance.

2.The methodology is a major strength, employing a two-stage least squares (2SLS) strategy to address endogeneity, supported by strong diagnostic tests. However, the justification for using geographic distance as an instrument for self-governance willingness, though standard, could be debated and would benefit from more robust theoretical underpinning.

We appreciate the reviewer’s careful observation. In the revision, we provide a fuller theoretical and empirical rationale for using geographic distance from each neighborhood to the provincial government’s administrative office as an instrument for self-governance willingness. Conceptually, distance operates as a proxy for institutional proximity and the intensity of top-down public-goods provision. Neighborhoods farther from the provincial seat attract less frequent attention, audit, and direct support from higher-level officials, thereby increasing reliance on local self-organization and, in turn, residents’ willingness to assume governance responsibilities. Conversely, neighborhoods near the provincial administrative office operate under the government’s immediate “shadow,” encouraging reliance on state provision and dampening bottom-up initiative. This mechanism is consistent with evidence from China showing that remoteness weakens top-down supervision and is associated with greater local irregularities (e.g., Li, 2019) and that administrative distance predicts the intensity of local public goods provision (e.g., Luo, 2006), as well as with broader work treating distance-to-capital as an exogenous proxy for government reach and influence.

We also address potential threats to the exclusion restriction—namely, that distance might capture broader spatial disparities unrelated to self-governance willingness. To mitigate this concern, our 2SLS specification includes district and sub-district (street) fixed effects, so identification is driven by within-district variation in proximity to the provincial hub, netting out urban-core/periphery and other coarse spatial differences. Under this design, geographic distance plausibly affects public service satisfaction only through its impact on self-governance willingness rather than through unobserved regional confounders. Finally, we report first-stage relevance and overidentification diagnostics (Kleibergen–Paap rk Wald F and Hansen J), which exceed conventional thresholds and do not reject instrument validity, respectively. Taken together, these theoretical, contextual, and econometric arguments substantiate distance as a credible instrument in our setting and reinforce the internal validity of our 2SLS estimates.

3.The results are clearly presented and well-interpreted. The mediation analysis using perceived neighborhood disparities adds depth, and the heterogeneity analysis across socioeconomic groups offers valuable insight. Nonetheless, more visualization (e.g., interaction plots or marginal effects) could enhance accessibility for non-technical readers.

Thank you for this constructive suggestion. To improve accessibility for non-technical readers, we added Fig. 2, a compact forest-plot visualization of the IV second stage point estimates with heteroskedasticity-robust 95% confidence intervals across all heterogeneity groups (top panel: self-governance willingness; bottom panel: participation efficacy). Markers denote coefficients, whiskers denote 95% intervals, and a vertical zero-line aids interpretation. The figure mirrors the subgroup patterns reported in Tables 8–11: intervals for low-income and urban-hukou residents exclude zero for both constructs; by housing area, willingness is significant only for smaller units while efficacy is positive in both and larger in magnitude for larger units; by ownership, willingness is significant in reform-era housing and efficacy is strongest in commercial housing. All estimates condition on the full covariate set with district and street fixed effects, and captions explain the plotting conventions.

We also added Fig. 3, a before–after panel of representative neighborhoods that visually conveys the scope and character of renewal in Hangzhou, providing intuitive context for the empirical results.

Taken together, these visualizations augment the mediation and heterogeneity analyses, transparently convey effect sizes and uncertainty, and respond to the request for clearer presentation while leaving the models and conclusions unchanged.

4.The discussion is theoretically grounded and policy-relevant, but limitations could be discussed more critically—particularly the reliance on cross-sectional data and potential measurement bias in self-reported variables. Future research directions could include experimental or longitudinal designs.

We appreciate this important feedback and have revised the Discussion to acknowledge our study’s limitations more explicitly and to outline concrete avenues for future research.

First, we now explicitly note that our data are cross-sectional (a one-time survey conducted in 2024), which inherently constrains our ability to make strong causal claims about changes over time. Although we address endogeneity concerns through an instrumental variable (IV) approach, the absence of longitudinal tracking prevents us from observing whether, and how, changes in self-governance willingness or participation efficacy within the same households translate into changes in satisfaction. We now emphasize this as a key limitation and propose that future research adopt longitudinal panel designs. For instance, a follow-up survey could repeatedly interview the same households—such as before and after a neighborhood renewal or participatory governance intervention—to capture temporal dynamics, reduce concerns about reverse causation, and assess the persistence of satisfaction effects over the long term.

Second, we acknowledge the potential for measurement bias in our self-reported variables. Both self-governance willingness and participation efficacy were measured through survey items (some binary, self-assessed responses), which may be subject to social desirability or recall bias. In designing the survey, we anticipated this concern and therefore conducted the data collection immediately after the completion of the neighborhood renewal projects to minimize recall errors and confounding influences. In the revised manuscript, we further recommend that future studies complement subjective survey measures with more objective indicators or mixed-methods approaches—for example, incorporating administrative records, direct observation of governance activities (e.g., meeting attendance logs, counts of resident proposals), or triangulating with qualitative interviews to validate and enrich self-reported responses.

Third, following the reviewer’s suggestion, we have expanded the Future Research Directions subsection to present concrete examples of experimental and quasi-experimental approaches. We agree that experimental designs can substantially strengthen causal inference. For example, in collaboration with local authorities, researchers could implement a randomized field experiment in which some neighborhoods are randomly assigned to receive enhanced participatory processes—such as additional pre-decision consultation meetings or structured feedback channels—while others follow the standard procedures. Comparing satisfaction outcomes across these groups would allow for a clean identification of participation effects, overcoming endogeneity concerns that observational studies cannot fully resolve. Evidence from other contexts supports this approach; for instance, a landmark field experiment in Indonesian villages found that randomly assigning communities to a more direct democratic process significantly increased resident satisfaction with local projects and perceived benefits. Building on such precedents, similar randomized interventions in Chinese urban neighborhoods could provide powerful tests of the mechanisms identified in our study.

Additionally, we suggest quasi-experimental strategies that exploit naturally occurring variation in policy implementation. For example, if certain districts adopt participatory budgeting or enhanced feedback systems earlier than others, a difference-in-differences design could be employed to compare changes in satisfaction between early adopters and later adopters. Similarly, the 2020 State Council directive mandating participatory processes in neighborhood renewal could serve as an exogenous policy shock, enabling comparisons of satisfaction before and after its implementation across different cities or districts.

By explicitly acknowledging these limitations and providing concrete methodological pathways—including longitudinal tracking, randomized field experiments, and quasi-experimental designs—we aim to fully address the reviewer’s concern. These additions not only strengthen the integrity of our discussion but also clearly chart a roadmap for extending the inquiry into participatory governance and public service satisfaction in future research.

Overall, this is a rigorous and significant contribution to urban governance literature, with room for modest enhancements.

**Response to Reviewer #2**

Reviewer #2: The manuscript presents a timely and empirically rich investigation into how self-governance willingness and participation efficacy shape residents’ satisfaction with urban public services in the context of neighborhood renewal in Hangzhou, China. Drawing upon a large-scale household survey (n=2,202) and employing a robust two-stage least squares (2SLS) estimation strategy to address endogeneity concerns, the authors offer a resident-centric perspective that advances the literature on participatory urban governance.

Strengths:

The study’s strengths lie in its comprehensive conceptual framework, methodological rigor, and clear policy relevance. The operationalization of self-governance willingness and participation efficacy as latent constructs derived from factor analysis is theoretically sound and empirically well-justified. Furthermore, the integration of perceived neighborhood disparity as a mediating mechanism—situated within relative deprivation and social equity theory—adds conceptual depth and analytical nuance. The heterogeneity analyses, disaggregated by income, hukou status, housing size, and ownership type, significantly enhance the manuscript’s empirical granularity and practical value.

The authors' use of distance to the provincial administrative office and residents’ policy awareness/community participation as instruments is innovative and justified, and the IV diagnostics demonstrate the robustness of the approach. The findings—that both self-governance willingness and participation efficacy positively influence public service satisfaction, though via distinct pathways—are both plausible and insightful.

Weaknesses and Limitations:

Despite these merits, the manuscript has several critical limitations:

1.Theoretical Overextension and Redundancy: While the authors ground their work in a broad swath of literature, the introduction and theory sections are overly dense and, at times, repetitive. The manuscript attempts to synthesize too many theoretical strands (e.g., participatory governance, relative deprivation, institutional design, procedural legitimacy) without fully integrating them into a coherent theoretical contribution. A more focused and critical engagement with the most relevant theoretical streams would enhance conceptual clarity.

We appreciate the reviewer’s insightful comment and have substantially revised the Introduction and Theoretical Framework to enhance conceptual clarity and focus. Specifically, (i) we consolidate overlapping discussions of participatory governance and procedural legitimacy into a single, coherent narrative; (ii) we streamline the treatment of relative deprivation by positioning it as the primary mechanism linking neighborhood disparities to perceived fairness, rather than combining multiple overlapping theories; and (iii) we remove or rephrase material not directly tied to our core hypotheses. The revised manuscript now advances a parsimonious causal pathway—resident agency (self-governance willingness; participation efficacy) → perceived neighborhood disparities → public service satisfaction—and aligns both hypotheses and empirical analyses to this framework. These revisions eliminate redundancy, sharpen theoretical boundaries, and foreground our distinctive contribution: situating residents’ participatory role as the central explanatory mechanism for variations in public service satisfaction.

2.Endogeneity Strategy Concerns: While the instrumental variables chosen are empirically tested, the theoretical justification for their exogeneity is somewhat weak. The assumption that distance to the provincial government’s office affects satisfaction only through self-governance willingness could be challenged, especially in a context where spatial inequality might independently influence satisfaction. A more robust defense of the exclusion restriction is warranted.

We appreciate the reviewer’s thoughtful concern and have strengthened both the theoretical and empirical justification for our IV strategy in the revised Methodology. We now define the instrument precisely as the distance from each neighborhood centroid to the provincial government’s administrative office, interpreted as a proxy for institutional proximity and the intensity of top-down public-goods provision in a hierarchical system. Neighborhoods farther from the provincial center have historically received fewer audits, site visits, and direct provincial projects, which increases reliance on local self-organization and, in turn, residents’ willingness to assume governance responsibilities. This mechanism is consistent with evidence from China that remoteness weakens top-down supervision and is associated with greater local irregularities (e.g., Li, 2019) and that administrative distance predicts the intensity of local public-goods provision (e.g., Luo, 2006), thereby supporting instrument relevance.

To address the possibility that distance captures broader spatial disparities unrelated to self-governance willingness, we bolster the exclusion restriction on several fronts. First, we include district and street (sub-district) fixed effects so that identification comes from within-district variation, netting out coarse spatial structure (e.g., urban core vs. periphery) and local service baselines. Second, we incorporate a rich set of covariates—Gender, Age, Education, Employment sector, Hukou status, Household size, and Annual household income—to absorb socioeconomic sorting correlated with location.

We further argue that the policy architecture in Hangzhou makes a direct pathway from provincial distance to satisfaction unlikely. The old-neighborhood renewal program is municipally funded and governed by a strong resident-voluntariness principle. As set out in the Implementation Plan for the Comprehensive Upgrading of Old Residential Communities issued by the General Office of the Hangzhou Municipal People’s Government, residents decide “whether to renovate, what to renovate, how to renovate, and how to manage,” and projects must satisfy the Property Law’s “double two-thirds” thresholds (eligibility and approval of the renovation scheme both ≥ 2/3 of owners). This concentrates decision authority and resourcing at the municipal/resident level, rendering any direct effect of provincial distance implausible beyond its indirect channel through self-governance willingness.

Finally, our IV diagnostics indicate that the instrument is empirically sound: the Kleibergen–Paap rk Wald F statistic exceeds conventional weak-instrument thresholds, and the Hansen J test does not reject the overidentifying restrictions. We nonetheless acknowledge that residual, unobserved spatial factors could in principle threaten the exclusion restriction and therefore state this limitation explicitly, interpreting the IV results with appropriate caution. Taken together, the articulated mechanism, institutional context, fixed-effects design with rich controls, and diagnostic evidence offer a robust and transparent defense of the exclusion restriction and reinforce the credibility of our endogeneity strategy.

3.Measurement Validity: The use of binary items to measure complex constructs such as “participation efficacy” and “self-governance willingness” raises questions about construct validity. While exploratory factor analysis supports the unidimensionality of the constructs, the dichotomous nature of the input variables limits the interpretive richness of the measures and may mask variation in resident attitudes.

We appreciate the reviewer’s concern that binary items may compress attitudinal variation. In the revision, we clarify that both self-governance willingness and participation efficacy are modeled as latent constructs estimated via exploratory factor analysis (EFA) from multiple, theory-driven binary indicators. For self-governance willingness, three responsibility-allocation items (decision-making, performance evaluation, property-management selection) load on a single factor (eigenvalue = 1.585; variance explained = 52.8%; KMO = 0.612; Bartlett’s χ² = 527.210, p < 0.01). For participation efficacy, three items (pre-decision consultation, process supervision, feedback responsiveness) load on a single factor (eigenvalue = 1.814; variance explained = 60.5%; KMO = 0.648; Bartlett’s χ² = 1027.199, p < 0.01). These diagnostics indicate a unidimensional factor structure and support construct validity. All models are estimated using continuous factor scores, which preserve gradations in the underlying attitudes despite dichotomous item responses.

To assess sensitivity to operationalization, we (i) reconstructed both constructs as additive indices and (ii) re-estimated the models using the original five-point Likert outcome for public-service satisfaction. Across these alternatives—and with the full covariate set plus district and street fixed effects—the substantive conclusions are unchanged (Table 7): self-governance willingness remains positive and significant (coefficients 0.754–0.980, p < 0.01), participation efficacy remains positive and significant (coefficients 0.362–0.501, p < 0.01), and perceived neighborhood disparity is consistently negatively associated with satisfaction.

Taken together—transparent construct design, a validated factor structure, continuous factor scoring, and convergent robustness evidence—these steps directly address the measurement-validity concern and demonstrate that the results are not artifacts of dichotomization or specific measurement choices.

4.Cross-sectional Design and Causality: As acknowledged by the authors, the cross-sectional nature of the data limits causal inference despite the use of IV estimation. Panel data or experimental interventions would provide stronger grounds for claiming causal effects. Furthermore, the assumed stability of attitudinal constructs like “willingness” and “efficacy” cannot be verified within the current design.

We agree that panel data or experiments would better capture dynamics. Our identification, however, does not rely on longitudinal stability of attitudes. The 2SLS design identifies contemporaneous local average treatment effects under standard IV assumptions—relevance, exclusion, and monotonicity—without requiring “willingness” or “efficacy” to be time-invariant. Monotonicity is plausible in our setting: greater institutional distance and stronger engagement cues (policy awareness, community activity) should not reduce self-governance willingness or participation efficacy among compliers.

Empirically, the IV strategy is well supported. Instruments are relevant (Kleibergen–Paap LM = 41.029, 55.254; KP Wald F = 78.196 for willingness, 29.013 for efficacy) and exogenous (Hansen J p = 0.753, 0.850). All models include the full covariate set plus district and street fixed effects (N = 2,202), so identification derives from within-district variation. First-stage relationships match theory (distance → willingness; policy awareness/community activity → efficacy); second-stage effects remain positive and significant ($\beta_{\mathrm{willingness}}$= 0.192, p < 0.05; $\beta_{\mathrm{efficacy}}$= 0.155, p < 0.01).

Findings are not artifacts of measurement choices. Re-estimations using additive indices for the constructs and the original five-point satisfaction outcome (Table 7) yield substantively unchanged results: willingness 0.754–0.980 (p < 0.01), efficacy 0.362–0.501 (p < 0.01), and perceived neighborhood disparity consistently negative. Mediation results (Tables 5–6) show coherent pathways (efficacy ↓ disparities → higher satisfaction; willingness exhibits a suppression via disparities). Because constructs enter as continuous factor scores, attitudinal gradations are preserved; any residual measurement error would bias estimates toward zero, making them conservative.

In sum, our conclusions do not depend on an assumption of attitudinal stability. They rest on a transparent IV design with strong first-stage strength, supportive overidentification tests, stringent fixed effects and controls, and robustness to alternative operationalizations—addressing the reviewer’s concern about causal inference in a cross-sectional design.

5.Overinterpretation of Subgroup Analyses: While the heterogeneity analyses are empirically interesting, the causal interpretation of subgroup effects may be overstated. The sample sizes in some subgroups (e.g., rural hukou holders, residents in reform-era housing) are relatively small, and confidence intervals are not reported. These limitations should be more explicitly acknowledged.

We appreciate the reviewer’s concern. First, we emphasize that causal identification is established in the baseline IV framework (Section 4.1; Table 4) and our causal language in the paper is anchored to those estimates. The subgroup analyses in Sections 5.1–5.4 are explicitly framed as exploratory/descriptive heterogeneity, not as separate causal claims. We have moderated wording throughout to avoid over-interpretation and to keep causal statements tied to the full-sample IV results with district and street fixed effects and the full covariate set.

Second, the subgroup samples are substantively sized and informative. By hukou: rural N = 387, urban N = 1,815 (Table 9). By income: low-income N = 966, middle/high-income N = 1,236 (Table 8). By housing area: ≤90 m² N = 1,579, >90 m² N = 623 (Table 10). By ownership: resettlement N = 425, commercial N = 657, reform-era N = 1,120 (Table 11). We now report standard errors and significance markers for all subgroup coefficients in Tables 8–11 (and maintain the same covariates and fixed effects as in the baseline), making clear which contrasts are statistically robust and where estimates are imprecise. Where coefficients are not significant (e.g., higher-income households for self-governance willingness), we state this plainly and refrain from strong claims.

Third, to address interval estimation and interpretability, we added Figure 2, a marginal-effects plot with 95% confidence intervals (e.g., participation efficacy × income). The figure clarifies which differences are statistically distinguishable (and where CIs overlap) and enhances accessibility for non-technical readers. Together with the updated tables, this presentation prevents overstatement and makes the uncertainty transparent.

In sum, the paper (i) confines causal interpretation to the identified IV results, (ii) documents adequate subgroup Ns and reports SEs/p-values for all subgroup estimates using consistent specifications, and (iii) visualizes marginal effects with CIs (Figure 2). These revisions directly address the concern about overinterpretation while preserving the substantive value of the heterogeneity evidence.

6.Lack of Critical Reflection on Participatory Governance in Authoritarian Contexts: The manuscript largely adopts a normative stance on participatory governance without engaging critically with the limitations of participation under authoritarian regimes. Issues such as tokenistic participation, constrained political space, or top-down orchestration of “voluntarism” are not discussed, which limits the critical depth of the paper.

We appreciate the reviewer’s concern. The core contribution of our study is not a general critique of participation under authoritarian regimes but an assessment of resident participation within a legally structured program—Hangzhou’s old-neighborhood renewal. In this program, although projects are government-funded, they cannot proceed without residents’ consent, as mandated by the Property Law’s “double two-thirds” rule (≥ 2/3 of owners to initiate and ≥ 2/3 to approve renovation plans). This procedural requirement ensures that participation is legally binding rather than symbolic, directly determining whether and how projects advance.

Empirically, our evidence indicates that participation in this context is substantive. Across the 36 surveyed neighborhoods, Figure 3 documents clear pre-/post-renewal improvements in amenities, public space, and service quality, which align with residents’ input shaping project outcomes. Econometric analyses further show that self-governance willingness and participation efficacy are significant predictors of satisfaction (Table 4), while mediation results demonstrate that efficacy operates partly by reducing perceived disparities (Tables 5–6). Robustness checks (Table 7) confirm that these findings are not artifacts of measurement or specification.

At the same time, we fully acknowledge that participatory spaces in authoritarian systems remain state-curated and politically bounded. Accordingly, we explicitly limit our causal interpretation to the IV-identified results within this service-delivery program, clarifying that our findings pertain to local governance of public services rather than broader political participation. Importantly, the Hangzhou case directly addresses the concern of “tokenism”: when participation is tied to statutory thresholds and financial disbursement, it carries genuine decision-making weight and produces observable improvements in community conditions.

In sum, while participatory governance under authoritarianism is inherently constrained, the legally mandated consent requirements of Hangzhou’s program and the documented substantive transformations (Figure 3) demonstrate that resident participation here is more than symbolic. We now emphasize this balance in both the Introduction and Discussion, ensuring that the manuscript reflects a critical, empirically grounded understanding of participatory governance in an authoritarian context.
