# Supplementary material for: How self-governance willingness and participation efficacy shape residents’ satisfaction with urban public services: Evidence from neighborhood renewal in Hangzhou, China
Source: PLoS One. 2026 Jan 23;21(1):e0341177. doi: 10.1371/journal.pone.0341177 (PMC12829847; doi:10.1371/journal.pone.0341177)
Supplement: Attachment — Submitted filename: Response to Reviewers-Round2.docx [file pone.0341177.s006.docx]

Remaining weaknesses (actionable)

Ethics committee identification still needed. The consent procedure is now clear, but the manuscript/cover materials (as shown) don’t name an IRB/ethics committee or provide approval/waiver identifiers. Add: committee name, approval number/date, and whether it covered verbal consent; or state that the committee granted a waiver/exemption, with details. This is the most important compliance gap to close.

Thank you for your thoughtful comment and for noting this important point regarding ethical approval. We confirm that this study received formal ethical clearance from the Medical Ethics Committee of Hangzhou City University prior to data collection (see appendix Approval Letter). The details are as follows:

- Ethics Committee: Medical Ethics Committee of Hangzhou City University
- Approval Reference: HZCU-2024-12
- Approval Date: September 02, 2024
- Project Title: 2024 Annual Resident Satisfaction Evaluation Report on the Renewal of Old Residential Communities in Hangzhou, China
- Principal Investigator: Yan Sun, School of Law, Hangzhou City University

The Committee determined that the research posed minimal risk to participants, involved adults aged 18 and above only, and collected no personally identifiable or sensitive information. It also granted explicit approval for the use of verbal informed consent in place of written consent. Prior to each interview, enumerators read a standardized consent script to participants, and verbal consent was documented on the questionnaire cover sheet. All data were de-identified at the point of collection and stored securely for academic use only.

We have now included this complete ethical approval statement, with the committee name and approval reference number, in the revised manuscript to ensure full compliance with PLOS ONE’s ethics and consent requirements.

Instrument exclusion restriction remains contestable. While the authors argue municipal governance and the “double two-thirds” rule localize decision authority, distance to the provincial government could still proxy unobserved spatial amenities or historic investment patterns affecting satisfaction directly. Consider (i) reporting robustness with additional spatial controls (e.g., distance to city center/rail hub, pre-renewal neighborhood quality), (ii) province-level road-network or historical connectivity controls, or (iii) a multi-instrument/over-ID setup using alternative plausibly exogenous instruments (e.g., historical administrative boundaries). At minimum, add a sensitivity table showing stability to richer spatial controls.

We sincerely thank the reviewer for this thoughtful comment regarding the exclusion restriction of the instrumental variable. In the original specification, we used the straight-line distance from each neighborhood’s centroid to the provincial government’s administrative office as the instrument for residents’ willingness to engage in self-governance. To address the reviewer’s concern that this variable might capture unobserved spatial amenities or historical investment patterns, we conducted additional robustness checks using two alternative, city-level instruments (see appendix S2 Table).

Specifically, Distance2 measures the distance from each neighborhood’s centroid to the Hangzhou Municipal Government office, and Distance3 measures the distance to the largest railway transportation hub in Hangzhou. These variables better capture intra-urban variation in administrative and infrastructural accessibility while mitigating potential confounding from provincial-level spatial heterogeneity.

As reported in Table S2, both alternative instruments remain strongly significant in the first stage, and the second-stage estimates for self-governance willingness remain stable in both magnitude and significance. The consistency of these results across specifications confirms the robustness of the findings and strengthens confidence in the validity of the exclusion restriction.

Cross-sectional design limits causal dynamics. The authors acknowledge this; the 2SLS yields contemporaneous LATEs. The Discussion could further emphasize that effects may incorporate short-term “halo” responses right after renewal and may attenuate over time; suggest a follow-up panel or pre/post cohort in future work.

We sincerely thank the reviewer for this valuable and insightful comment. We fully agree that the cross-sectional design constrains the identification of dynamic causal relationships and that the 2SLS results should be understood as contemporaneous local average treatment effects (LATEs). In response, we have revised the Limitations section (Section 6.2) to explicitly discuss the possibility of short-term “halo” responses immediately after the completion of renewal projects and to note that such effects may weaken over time. We also suggest that future research employ longitudinal or pre/post cohort designs to capture temporal dynamics and evaluate the durability of these effects. These additions clarify the interpretive scope of our findings and reflect the reviewer’s thoughtful recommendation.

Self-report bias and common-method variance. The survey timing and factor modeling help, but the paper could add explicit checks (e.g., negative-control items or administrative corroboration of renewal features) or at least a brief CMV diagnostic and discussion. (Not a blocker, but strengthens credibility.)

We sincerely appreciate the reviewer’s insightful suggestion regarding potential self-report bias and common-method variance (CMV). To address this concern, we have added both a diagnostic test and an explicit discussion in the revised manuscript(see appendix S1 Table).

First, we conducted Harman’s single-factor test, which showed that the first factor explained 30.4% of the total variance—well below the 40% threshold commonly used to indicate serious CMV risk (see S1 Table). The result suggests that common-method bias is unlikely to substantially affect our findings.

Second, to further reduce potential self-report bias, several key renewal indicators—including the completion progress, participation procedures, and voting records under the legally binding “double two-thirds” rule were cross-validated using administrative documentation obtained from the Hangzhou Urban Renewal Office. This triangulation provides an additional layer of credibility to the survey data. In addition, we have explicitly acknowledged in the Limitations section (Section 6.2) that self-reported measures of governance willingness and participation efficacy may still be subject to social desirability and recall bias, and we recommend that future research incorporate more objective indicators (e.g., meeting attendance or proposal submission records) and mixed-methods approaches to enhance measurement validity.

Finally, these revisions, together with the expanded discussion of data limitations in the Discussion and Limitations sections, collectively improve methodological transparency and reinforce the overall robustness and credibility of the study.

Minor but visible production issues. Remove residual Word markup (“Formatted: Font: Not Bold”) and ensure figure/table cross-references are clean and consistent before acceptance.

Thank you for your careful observation. In accordance with the editorial requirements for the first-round revised manuscript, we were asked to provide both a clean version and a tracked-changes version of the submission. The visible notes such as “Formatted: Font: Not Bold” appear only in the tracked-changes version to indicate formatting edits made during revision. These are automatically generated by Microsoft Word’s Track Changes function and are not part of the final layout. The clean version, which has been fully aligned with the PLOS ONE template, contains no such formatting artifacts. We have also carefully rechecked all figure and table cross-references to ensure that they are fully clean, consistent, and ready for production.

Verdict

Substantial improvement; nearly there. The R1 addresses most editorial and reviewer requests convincingly: funding/role statements, data availability, theory sharpening, measurement transparency, IV reporting, mediation framing, and heterogeneity presentation are all improved. The one material compliance gap is the absence (in the provided materials) of an explicit IRB/ethics committee name and approval/waiver information. Add that, plus a short robustness note on the IV exclusion restriction and a final formatting pass, and the manuscript would be ready for consideration.

If you’d like, I can draft a short author checklist paragraph that you can paste into your cover note summarizing these last fixes.

Reviewer #3: The authors have satisfactorily addressed all comments raised in the previous round of review. The manuscript is now technically sound, with robust methodology and data that clearly support the stated conclusions. The statistical analyses have been conducted with rigor and are appropriate for the research objectives. All underlying data have been made fully available, ensuring transparency and reproducibility. The manuscript is well-organized, clearly written in standard academic English, and presented in an intelligible manner. Overall, the revisions have strengthened the quality of the paper, and it is now suitable for publication.

We sincerely appreciate the reviewer’s thoughtful and encouraging comments. We are deeply grateful for the constructive feedback provided throughout the review process, which has been invaluable in refining the rigor, clarity, and overall quality of the manuscript. We are pleased that the revised version is now recognized as technically sound, methodologically robust, and well organized. We thank the reviewer and the editorial team for their time, expertise, and kind support, which have greatly contributed to improving this work.
